# Metadata and Reuse: Antidotes to Information Entropy

**DOI:** 10.1016/j.patter.2020.100004

**Published:** 2020-04-10

**Authors:** Ted Habermann

**Affiliations:** 1 Metadata Game Changers, Boulder, CO 80304, USA

**Keywords:** metadata, FAIR data, data stewardship, data reuse, data repository, information entropy, interoperability

## Abstract

Entropy is the natural tendency for decline toward disorder over time. Information entropy is the decline in data, information, and understanding that occurs after data are used and results are published. As time passes, the information slowly fades into obscurity. Data discovery is not enough to slow this process. High-quality metadata that support understanding and reuse and cross domains are a critical antidote to information entropy, particularly as it supports reuse of the data—adding to community knowledge and wisdom. Ensuring the creation and preservation of these metadata is a responsibility shared across the entire data life cycle from creation through analysis and publication to archiving and reuse. Repositories can play an important role in this process by augmenting metadata through time with persistent identifiers and connections they facilitate. Data providers need to work with repositories to encourage metadata evolution as new capabilities and connections emerge.

## The Bigger Picture

Reusing data requires understanding, trust, and a community that values complete metadata and actively participates in the long-term knowledge building process. Participants include authors who document their data well using generic metadata standards and accessible conventions, researchers who review manuscripts and data with reuse in mind, and readers who keep an eye out for well-documented data and then use that data to build community knowledge and wisdom. Repositories play a critical active stewardship role in this process, augmenting and managing metadata that connect researchers and research objects across the community. This participation extends the data life cycle, broadens impact, and slows information entropy.

## Teaser

Are data from your published work suffering from information entropy since they were published? Are they experiencing a slow decrease in impact because other researchers cannot understand or trust them? High-quality metadata, persistent identifiers, and active repository partnerships might help revive them and ensure their role in building community understanding and wisdom.

## Main Text

**Figure 1:**
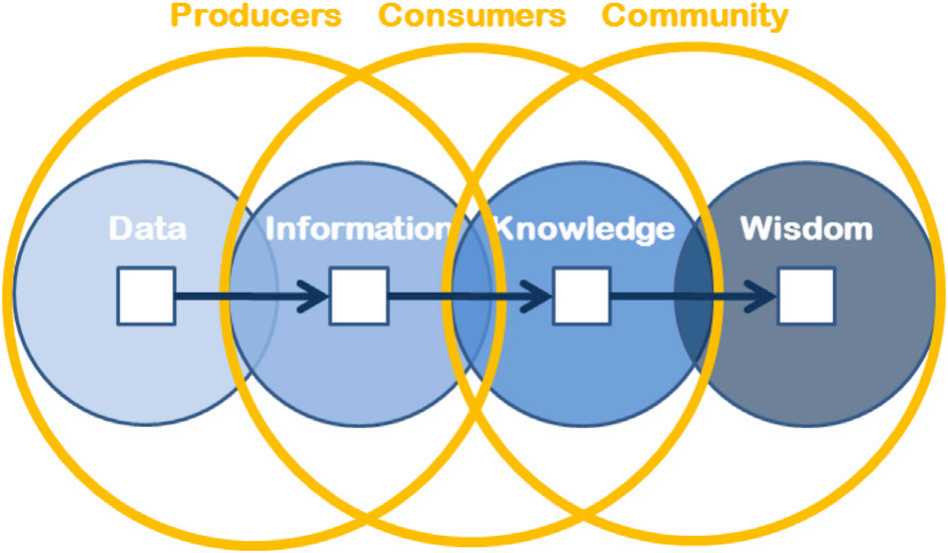
The Continuum of Understanding Framework for Describing How Data Become Wisdom

What happens to data as they move into the future? An idealized answer can be built on the concept of the Continuum of Understanding (Figure 1) originally described by Cleveland^1^ and elaborated by Shedroff.^2^ The continuum has four stages: data, information, knowledge, and wisdom. Data are observations and model results that are collected from the world around us. They are numbers that characterize some phenomena but, by themselves, they are not very useful. Structure, context, and organization are added to create information that can be shared and absorbed by others. Individuals create knowledge as they consume information from multiple sources and merge it with their experience. The knowledge stage of the continuum is where most human discourse happens. People share the knowledge that they have gained and present their points of view (context). This discourse hopefully leads to wisdom, i.e., community understanding of the object of study based ultimately on the original data.

Groups that participate in this process vary along the continuum. Researchers (data producers) formulate scientific questions and collect data to answer them. They add structure and context to the observations in the form of metadata, presentations, and papers, and share the resulting information with consumers. They use software developed by other researchers who may, or may not, be on the project team. In some cases, they understand the intricate details of that software and the assumptions relevant to its use and interpretation of the results. In others, they may not. Data Centers, repositories, and data curators play an important and useful mediation role in facilitating the data/information sharing process and broadening the community of consumers. Finally, in the wisdom part of the continuum, community contributes as consumers interact with each other. Knowledge is shared, and community wisdom is constructed.

**Figure 2:**
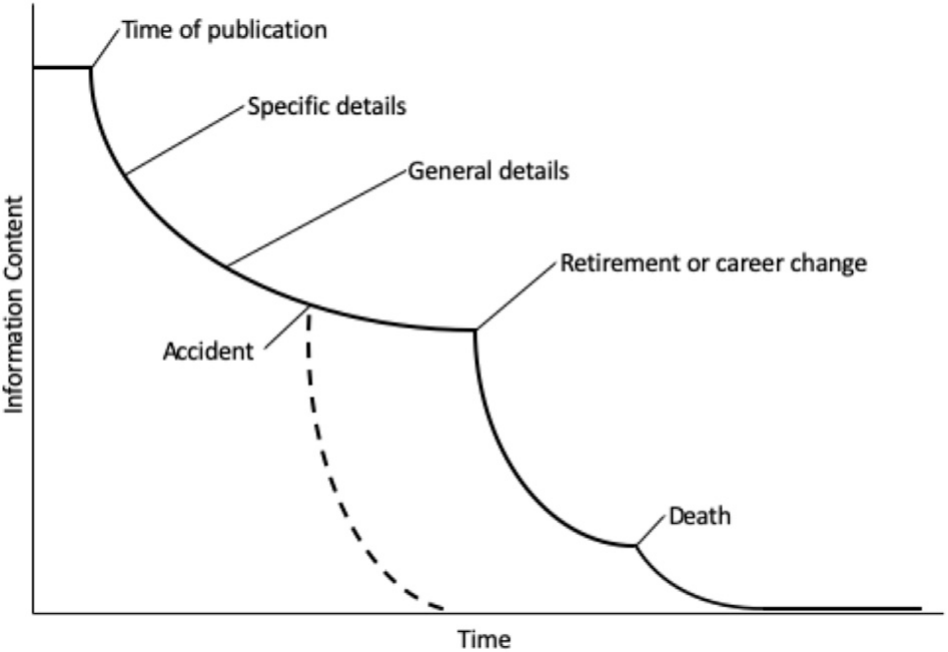
Information Entropy Is the Degradation of Information without Metadata and Curation See Michener et al.^3^ for more information.

An alternative picture is presented by Michener et al.^3^ who applied the Shannon^4^ concept of “information entropy” (Figure 2) to describe the loss of information content over time due to degradation of the raw data or metadata. The information content peaks at the time of publication and then falls off over time in a number of steps. This is the fate of data without curation and preservation contributions of the researchers, software developers, data curators, and users mentioned above.

Michener et al. described the critical role that metadata play in slowing or preventing information entropy. Some people describe metadata as “data about data.” Michener et al. provide a more informative definition: “metadata are the information necessary to understand and effectively use data, including documentation of the dataset contents, context, quality, structure, and accessibility.” The definition that I have relied on is that documentation is all of the information, in any format, required to reproduce a result, and that metadata are the structured and standard part of that documentation. This emphasizes the role of metadata in data sharing (standard) and machine readability (structure).

**Figure 3:**
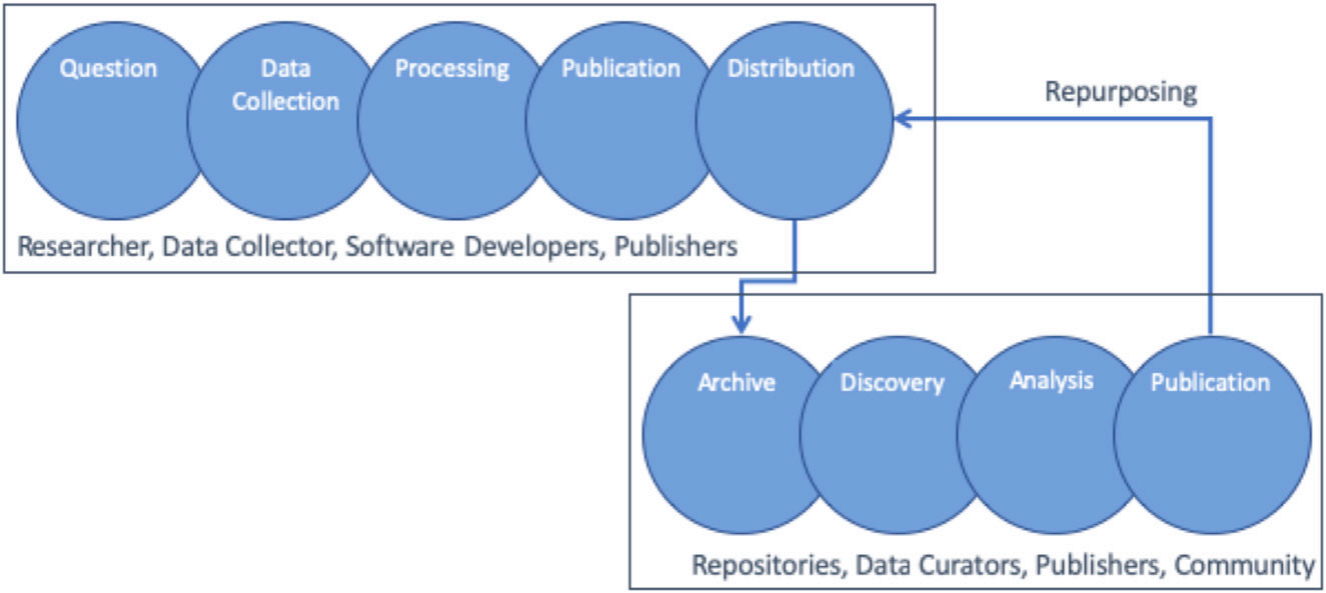
Schematic of the Data Life Cycle

Given these more informative and comprehensive definitions of metadata, the obvious question is: who creates and maintains the metadata necessary to avoid information entropy? The right answer has to be everyone involved with creating, processing, preserving, publishing, and using the data. Many people use the concept of the data life cycle to frame discussions of the steps and processes that occur over the life of data. Figure 3 shows one version of the data life cycle and identifies groups of people who contribute metadata at various phases in the cycle. There is a clear division of labor here. People in the first group contribute metadata because they contributed to the creation of the data, i.e., they “know” the data. People in the second group contribute metadata because they are data users or “know” the users. By consuming the original data and creating value-added products, they develop additional knowledge and understanding of the data and the objects it characterizes.

This division of labor in Figure 3 has been recognized before. Lyon^5^ described roles, rights, responsibilities, and relationships for scientists, users, and organizations involved in the data life cycle. Two of the roles described are particularly relevant to this discussion. Scientists have the role “work up data for use by others” and Data Centers have the role of “providing tools for re-use of data.” Wallis et al.^6^ pointed out that both of these roles can be expensive and time-consuming and that it is hard to justify that work without knowing that data will be reused or what it will be reused for. Mayernik^7^ (2011), also pointed out that the lack of understanding of or knowledge of future users made creation of metadata-for-data-sharing difficult.

Lyon identified “meet standards for good practice” as a responsibility of players in both segments of the data life cycle. Creating and maintaining data and metadata that are compliant with standards can be a significant portion of the increased effort associated with data sharing and reuse. The scope of the standards is also in this equation: community standards may be closer to the working practice/needs in a particular domain, i.e., easier in the short term, but they can also limit the scope of reuse. This is reflected in the observation that much of the data that are shared on project, laboratory, or program websites, rather than in repositories, do not have the metadata that are required for discovery or reuse outside of a small group of trusted colleagues.^6^ Is it possible to increase the benefit of standards while minimizing the effort required?

### Flexible Conceptual Standards that Cross Domains

The data life cycle in Figure 3 covers a broad range of activities and long time periods. This makes it almost certain that it covers a broad range of technologies and tools and potentially a broad range of domains. This diversity presents a significant challenge to metadata standardization processes. Can standards form a foundation serving cross-domains needs while also providing for specialized domain-specific needs? Metadata standards for geographic data developed by ISO Technical Committee 211^8^ have several characteristics that might help address this need for cross-domain standardization. Communities developing or selecting metadata conventions or standards could benefit from emulating these characteristics.

Historically, a simple statement that a particular metadata standard does not “fit” a particular type of data is sufficient to justify not using that standard, or even constructing a new one. The ISO 19115 metadata standard addresses this challenge by defining a standard mechanism for adding extensions. This is an important response to the “does not fit” justification and an important mechanism for increasing the breadth of the community that can take advantage of the generic aspects of the metadata standard. More importantly, it increases the breadth of the community that can discover, use, and understand the data, facilitating the creation of knowledge and wisdom beyond the original intended scope of the data and information.

This two-layer metadata model, a standard foundation with domain-specific extensions, fits well into the context of the FAIR Principles for data and metadata.^9^ Metadata concepts that support Findability, e.g., title, author, keywords, abstract, temporal, and spatial extent, are shared across many domains and are required in many metadata dialects used in repositories that support data discovery.^10^ Access is also covered in many repositories because of the ubiquitous landing pages associated with DOIs and other identifiers.

Metadata elements that support tnteroperability and reuse tend to be more specific, e.g., standard data formats and parameter names, data quality measures and results, community vocabularies, and therefore less likely to be included directly in generic standards. These elements appear in the domain-specific vocabularies and extensions described above.

Another aspect of the ISO 191∗ family of standards is a grounding in a general conceptual model in Universal Modeling Language (UML). These models provide starting patterns for documenting many important aspects of data, including discovery, data quality, data services, data lineage, constraints, spatial and temporal extents, and others. Documentation patterns defined at a conceptual level can be shared across many domains and represented in different ways (e.g., XML, JSON, RDF), providing resilience and utility through time.

An example that demonstrates this conceptual approach and the breadth of the ISO standard is the model for user feedback, an important element in the data life cycle illustrated above. ISO 19115-1^11^ includes a UML class that includes descriptions of what a specific user tried to do with the data, when they tried to do it, limitations that they identified in the process, a link to a list of issues identified with the data through time, and a response from the data provider. Users and reusers have always been able to identify problems and limitations of data. These metadata connect those discoveries to the on-going discussion and improvement of the dataset. This extends the metadata creation process throughout the data life cycle and extends the range of the feedback loop to include future users who have access to the growing metadata and documentation collection.

### Repository Roles in Metadata Augmentation

Figure 3 divides the data life cycle into a segment led by researchers and a segment led by repositories. A study of how researchers share data^6^ showed that only a small number of scientists use repositories to share data. In other words, the second part of the data life cycle does not exist for many datasets because the perceived benefits of repositories are small relative to requirements and burdens of data preparation and deposition. Can repositories change this equation by creating metadata that provide new benefits to data providers?

Metrics related to citations have been collected for many years as a way to measure the impact and influence of articles, researchers, institutions, journals, and publishers. These metrics are metadata and, despite a number of challenges,^12^ provide information that users find useful. More recently, other metrics have been developed based on a broader set of observations^13^ and applied to data.^14^^,^^15^ These metrics are well-established and accepted examples of repositories augmenting metadata. Are there more opportunities for metadata added by repositories in the second segment of the data life cycle?

The user feedback metadata described above may be one such opportunity. Repositories can take advantage of user feedback metadata and help create wisdom by facilitating and mediating the flow of information between users and data providers. This element of community and wisdom building has emerged as an integral part of the culture of the World Wide Web, spanning the gamut from tagging systems through hashtags and social networks to wikis as mechanisms for collecting and sharing information from users about data. It includes the ideas and methods of citizen science and crowdsourcing to move from data to information and toward knowledge and wisdom. It will be an important way of integrating community into on-going metadata, documentation and data reuse efforts.

Repositories (and data publishers) have a second important role in accelerating adoption and implementation of new ideas, needs, and capabilities as they emerge. An important example is identifiers that remain functional throughout the data life cycle, i.e., persistent identifiers (PIDs). These identifiers are critical elements of several of the FAIR data management principles,^9^ enabling qualified connections between people, organizations, papers, datasets, software, and other research objects and supporting access to and retrieval of all identified research objects.

The benefits of these connections are clear,^16^^,^^17^ but the identifiers must be included in the metadata to realize them. Getting PIDs into metadata requires concerted effort on many fronts. Good examples and the workflows that create them need to be identified and socialized. The Crossref Participation Reports^18^ were designed, in part, to increase awareness of identifiers in Crossref metadata and services they enable. These data show increases in utilization of many connections^19^ but overall adoption rates remain in the 10%–20% range.

Increasing identifier adoption requires (1) evolving metadata schemes to include all types of identifiers^20^ and (2) working with providers and users to develop trusted workflows for automatically augmenting metadata with persistent identifiers. Tracking the provenance of these workflows and the responsible parties will be critical^21^ to building trust in these processes.

### Conclusion

**Figure 4:**
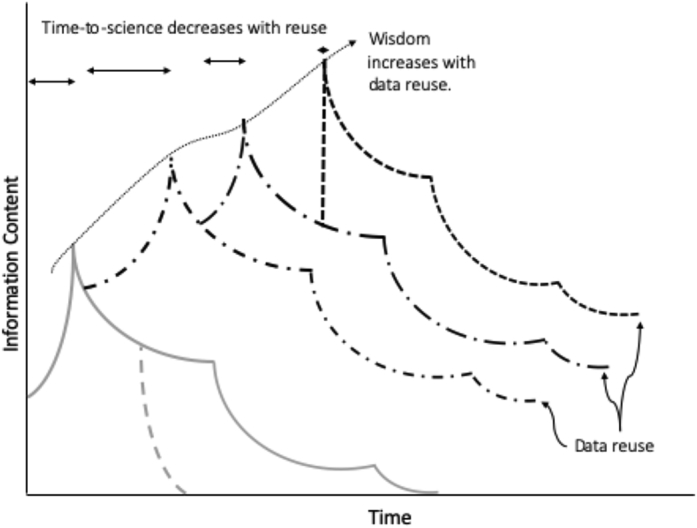
Negentropy Adds Information Content to Data through Time by Reuse in Multiple Communities

The Continuum of Understanding provides an optimistic vision whereby data + community = wisdom. Warnings about “information entropy” add metadata to the equation: data + metadata + community = wisdom. Reuse is another positive element that introduces extension of information and knowledge into new, and potentially unexpected directions and communities, broadening impact and information content. This is the opposite of “information entropy” termed “information negentropy” and illustrated in Figure 4. In this schematic, each data entropy curve added to the original corresponds to a reuse of the data in a different community. Each reuse adds valuable information content and contributes to broader wisdom spread over multiple communities.

Figure 4 shows phases of data > information > knowledge as increases in information content before publication peaks. This corresponds to the first phase of the data life cycle shown in Figure 3. This is the “time-to-science” for each data use. Anticipated long times-to-science can be strong obstacles to reuse. Complete, high-quality input from researchers, data collectors, software developers, and publishers during the initial data collection and development can help address this obstacle. This schematic shows time-to-science decreasing through multiple reuse cycles, assuming that the metadata associated with the data and trust in the data increases through time. This optimistic scenario depends on the metadata and PIDs contributed to the system by repositories, data curators, publishers, and users during subsequent cycles.

Coupling extensible metadata standards with the broad adoption of PIDs simultaneously slows or even precludes data entropy and accelerates progress along the Continuum of Understanding. The community of authors, reviewers, editors, and readers of *Patterns* can make important contributions to building wisdom during initial data development and subsequent reuse cycles by:

• Identifying people, organizations, data, software, instruments, and other research objects with persistent identifiers.
• Describing data and processing with metadata that facilitates understanding and trust.
• Connect to other data and results with links.
• Minimize time-to-science during subsequent reuse cycles and add information during those cycles to further decrease time-to-science.

The foundation is strong, the future looks exciting. Let's do it!
